# Are Survey-Based Estimates of the Burden of Drug Resistant TB Too Low? Insight from a Simulation Study

**DOI:** 10.1371/journal.pone.0002363

**Published:** 2008-06-04

**Authors:** Ted Cohen, Caroline Colijn, Bryson Finklea, Abigail Wright, Matteo Zignol, Alexander Pym, Megan Murray

**Affiliations:** 1 Division of Social Medicine and Health Inequalities, Brigham and Women's Hospital, Boston, Massachusetts, United States of America; 2 Department of Epidemiology, Harvard School of Public Health, Boston, Massachusetts, United States of America; 3 STOP TB Department, World Health Organization, Geneva, Switzerland; 4 Clinical and Biomedical TB Research Unit, South African MRC, Durban, South Africa; 5 Division of Infectious Diseases, Massachusetts General Hospital, Boston, Massachusetts, United States of America; The National Institute for Public Health and the Environment, Netherlands

## Abstract

**Background:**

The emergence of tuberculosis resistant to multiple first- and second-line antibiotics poses challenges to a global control strategy that relies on standard drug treatment regimens. Highly drug-resistant strains of *Mycobacterium tuberculosis* have been implicated in outbreaks and have been found throughout the world; a comprehensive understanding the magnitude of this threat requires an accurate assessment of the worldwide burden of resistance. Unfortunately, in many settings where resistance is emerging, laboratory capacity is limited and estimates of the burden of resistance are obtained by performing drug sensitivity testing on a sample of incident cases rather than through the use of routine surveillance.

**Methodology/Principal Findings:**

Using an individual-based dynamic tuberculosis model to simulate surveillance strategies for drug resistance, we found that current surveys may underestimate the total burden of resistant tuberculosis because cases of acquired resistance are undercounted and resistance among prevalent cases is not assessed. We explored how this bias is affected by the maturity of the epidemic and by the introduction of interventions that target the emergence and spread of resistant tuberculosis.

**Conclusions:**

Estimates of drug resistant tuberculosis based on samples of incident cases should be viewed as a lower bound of the total burden of resistance.

## Introduction

Extensively drug-resistant tuberculosis (XDR TB) has been documented in forty-seven countries on six continents [1]; XDR TB is characterized by resistance to the two most important anti-TB drugs (isoniazid and rifampin) plus additional resistance to at least one fluoroquinolone and one injectable antibiotic [2]. While the most TB cases worldwide can be effectively treated with standard drug regimens, the unchecked emergence of resistance may compromise the effectiveness of global disease control strategies [3]. In order to assess the need for and design control strategies that can address the threat of emerging resistance, public health practitioners need an accurate assessment of the burden of drug-resistant tuberculosis.

Unfortunately, in many high-burden settings, the paucity of laboratories equipped to perform routine culture and drug-susceptibility testing hampers efforts to document the extent of the problem. In these resource-constrained settings, estimates of resistance are determined through periodic surveys of incident (*i.e.* presenting) TB cases rather than through ongoing surveillance. The existing guidelines for the design of these drug-resistance surveys emphasize that valid inference requires that individuals included in the sample must be randomly selected from the total population of incident tuberculosis cases in the region under evaluation. Additionally, these guidelines suggest that individuals with first-time tuberculosis should be analyzed separately from those with recurrent disease, since those with recurrent disease are more likely to harbor resistant strains [4].

While this type of survey allows the estimation of resistance among newly-occurring or recurring cases of TB, we suggest that incidence-based samples may underestimate the total burden of drug-resistant tuberculosis in a community. First, since these surveys are conducted among individuals with a new or recurrent diagnosis of tuberculosis, patients who acquire drug resistance while enrolled in a treatment program may not be re-registered as recurrent cases, and thus would be less likely to be included in the study sample. Second, we propose that the total burden of resistance should also reflect the extent of resistance among prevalent (*i.e.* extant) TB in a community, since these individuals are the potential source cases for transmitted resistance, and represent the current resource demand for second- or third-line antibiotics. Individuals with drug-resistant disease are likely to experience longer durations of illness, since they will respond less favorably to standard drug regimens; therefore, the proportion of prevalent cases that is resistant is likely to exceed the proportion of incident cases that is resistant.

In this paper we describe a simple dynamic model of TB transmission designed to simulate the performance of incidence-based sampling methods. We use this model to examine the validity of sample-based estimates of the total burden of drug resistance and explore how the magnitude of bias in these sample-based estimates is affected by the maturity of the drug-resistant tuberculosis epidemic and the implementation of different types of public health interventions.

## Methods

### Tuberculosis model

#### Generation of transmission networks

We simulate the natural history and transmission of drug-sensitive and drug-resistant strains of tuberculosis over a simple idealized contact network in which transmission of disease is more likely to occur between individuals who are in close socio-spatial proximity. These networks are not intended to capture the contact structure of any particular population, rather they aim to represent the concept that individuals are more likely to contact others who reside and circulate within their social context than other individuals randomly selected from the population. The generation of these networks is described fully in a previous manuscript [5]; briefly, following the method of Read & Keeling [6], we generate a population in which each individual is placed at random on a square patch at a constant average density. We depict contacts between these individuals as edges connecting vertices, with the presence of an edge signifying sufficient contact for transmission of disease. The probability of an edge between two individuals decreases as the distance between them increases, such that infection is transmitted preferentially to individuals in the proximity of an infectious case. Thus, individuals located nearest each other on the network can be thought of as family members, while those slightly further away may be neighbors, friends, or other social contacts. We specify how “clustered” the network is by setting a single parameter *D* (lower *D*  =  higher cliquishness) and assign an average number of contacts (*i.e.* degree) by specifying a second parameter *n.* We use the relationship
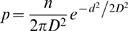
such that for any two vertices separated by a distance *d*, the probability of an edge linking them is equal to *p*.

#### Modeling the natural history and transmission of TB on the network

**Figure 1:**
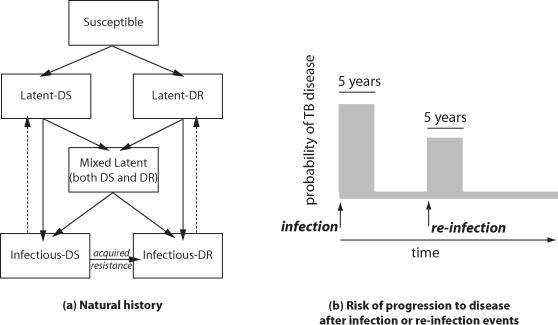
A) Natural history of TB infection. Individuals are born susceptible to infection by either DS or DR TB. Latently infected individuals can be re-infected by any circulating strain; if re-infected by the other strain, they transition to a state of mixed latency. The risk of progression to TB disease from latently infected states depends on the time since the most recent infection event. DR TB first appears through the acquired route and can then be transmitted. Individuals in the infectious states suffer a higher disease-specific risk of death, and those with DR TB are less likely to be effectively treated by antibiotics. B) The probability of progression to disease is dependent on the time elapsed since infection. Individuals who are recently infected or re-infected (within 5 years of such events) experience an increased risk of progression to active TB disease compared with individuals who were infected or re-infected at more distant times.

We model the natural history of tuberculosis using a modified susceptible-latent-infectious-recovered (SEIR) model where each individual is born susceptible (S) and has a probability (φ) of being infected during each month of contact with one or more infectious individuals. Upon infection, an individual transitions to a state of latent infection (E) from which he or she may progress to active tuberculosis disease (I) (Fig. 1A). The probability of progression from latent infection to disease is dependent on the duration of infection; here, the risk of progression is greatest within five years of an infection event, and is much reduced after this period has elapsed [7], [8]. A latently infected individual may also be re-infected by a second circulating strain of *M. tuberculosis*, though latently infected individuals retain partial immunity to re-infection (Fig. 1B). Individuals recovering from tuberculosis, either through treatment or self-recovery, transition back to a state of latency from which they have only a small probability of progression.

**Figure 2:**
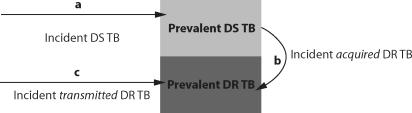
Mechanisms of resistance. Incidence of drug-sensitive TB (DS TB) contributes to the pool of prevalent DS TB (arrow a). Incidence of drug-resistant TB (DR TB) occurs either through acquired drug resistance (arrow b) or through transmitted/primary resistance (arrow c).

We include two strain phenotypes in this model, one which we designate “drug sensitive” (DS) and one which we designate “drug resistant” (DR). These broad categorizations, and the associated strain-specific parameter values, are intended to reflect the fact that some strains of tuberculosis respond well to standard multiple-drug chemotherapeutic regimens (e.g. strains without any resistance or with resistance to only single drugs in the regimen), while other strains that are resistant to more than one drug (especially those with resistance to the two most powerful drugs, isoniazid and rifampin) respond relatively poorly to standard treatment regimens [9]. Mycobacterial resistance to anti-tuberculosis drugs initially emerges within treated hosts by the selection of rare, sporadically-occurring mutants under conditions of inadequate chemotherapy (acquired drug resistance; Fig. 2, arrow b). Once resistant strains have emerged, these strains can be transmitted to others (transmitted, or primary, resistance; Fig. 2, arrow c). We model the emergence of DR assuming that resistance first occurs in a fraction of individuals who are on treatment for active disease, and may then be transmitted to their contacts. For simplicity, we model the acquisition of the DR phenotype as a single-step process, while in reality resistance to multiple antibiotics is caused by sequentially-occurring mutations [10], [11], [12]. Since individuals with DR disease are less responsive to standard therapeutic regimens, they will, on average, experience a longer duration of infectiousness, and therefore also a higher case fatality.

We assume that most resistance-conferring mutations disrupt gene function and thus exact a fitness cost which may reduce the pathogen's ability to be transmitted and/or to cause disease. Previous models have demonstrated that the relative fitness of DR strains compared with DS strains is a key determinant of the trajectory of DR tuberculosis epidemics [13], [14], [15], and the mean relative fitness of DR strains may increase over time as more fit DR strains are preferentially transmitted [16]. Studies suggest that while laboratory-derived drug-resistant bacterial isolates usually have substantial fitness deficits, resistant strains collected from clinical specimens may not be similarly impaired [17]–[24]. For the baseline simulations we present, we assume that the DR strain has a fixed moderate fitness cost which reduces both the probability of transmission and the probability of progression after infection by 20% each; in further analyses, we discuss how an increasing mean relative fitness among DR strains over the course of a drug resistant TB epidemic would affect our results.

We calculate each individual's per month probability of infection with either the DS or the DR strain (φ_DS_, φ_DR_) by first considering the probability of *not* being infected (∼φ_DS_, ∼φ_DR_). Given that τ_DS_ and τ_DR_ are the infectiousness per month of drug-sensitive and drug-resistant infectious contacts, respectively, and that k_DS_ and k_DR_ are the number of each type of contact, then ∼φ_DS_ = (1−τ_DS_) ^kDS^ and ∼φ_DR_ = (1−τ_DR_) ^kDR^. Thus, the total probability of being infected with either strain is equal to one minus the product of these two, that is, φ = 1−[(∼φ_DS_)(∼φ_DR_)]. Since we allow that only one infection event can occur per time step, the final probabilities of infection are the products of the total probability and the proportion of neighbors infectious with each strain:
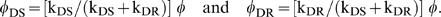
For individuals in the latently infected state, who remain only partially susceptible to re-infection, these infection probabilities are reduced by the immunity factor.

Recent investigations suggest that concurrent infection with multiple strains is possible and may not be unusual in high-disease-burden settings, where the force of infection is large [25]–[31]. In this model, we include a state of mixed latency to reflect the fact that some individuals may harbor infection with both DR and DS strains; therefore, each individual in the model is in one of six states: susceptible (S), drug-sensitive latent infection (E_DS_), drug-resistant latent infection (E_DS_), mixed latent infection (E_M_), active drug-sensitive tuberculosis (I_DS_), or active drug-resistant tuberculosis (I_DR_). Parameter values (Table S1, Figure S1) and additional modeling details (Supplement S1, Figure S2), are provided in the supplementary material.

### Simulating drug-resistance surveys

To illustrate the performance of drug-resistance surveys conducted during tuberculosis epidemics, we compare the estimated burden of drug resistance obtained from incidence-based surveys to the actual proportion of drug resistance among (*i*) incident and (*ii*) prevalent cases of disease as drug resistant TB emerges in the population. We use the emerging epidemic of drug-resistant TB in the Russian Federation between 1990 and 2003 as a guideline to simulate growing epidemic of multidrug resistant TB (MDR TB–resistance to at least isoniazid and rifampin), choosing transmission parameters accordingly and taking other parameters from the literature (Supplement S1, Table S1). The key features of this epidemic are that there is a rising burden of TB as well as drug resistant TB and that it is not heavily influenced by an HIV co-epidemic. We note that our model should not be viewed as a model for the tuberculosis epidemic in a specific geographic location, rather we use it as a general example of an emerging drug resistant TB epidemic in the absence of HIV. During this time period, the estimated total TB incidence rose from 48 to 112 cases per 100,000 per year [32]. Trends in the proportion of MDR among incident cases were estimated using a weighted average of results from previously-treated and never-treated patients surveyed in 1998, 2000, 2001, and 2002. Over this span, the proportion of incident cases that were highly resistant rose from approximately 9 to 19% [33]. We then extend the simulations for an additional 10 years to permit us to explore changes in the performance of surveillance methods over longer time spans and in the presence of different control strategies.

While parameters governing the topology of the contact network for TB are not known, the network approach adopted here allows individuals to be more likely to transmit disease to close social contacts than to random individuals, and allows these close social contacts to be in contact with each other (clustering); again, the network is not intended to duplicate the contact structure of a particular community. For the simulations we present here, we use networks with 100,000 vertices and moderate locality parameter (*D* = 5) and mean degree (*n* = 15). Importantly, the qualitative results we present are not sensitive to these choices, and in fact hold over a range of parameter choices leading to growing drug-resistant TB epidemics.

In our model, individuals are counted as incident drug resistant cases in the simulated surveys if they have resistant disease at the time they are diagnosed as TB cases; thus, DR TB cases due to primary transmission of resistance and DR TB cases who have acquired resistance during a previous course of therapy and subsequently re-present with resistant disease will be identified. Conversely, we have assumed that individuals who present with drug sensitive disease and acquire resistance during the current course of therapy will not be identified as resistant cases in these surveys since at the time of diagnosis they would not have been identified as DR TB cases. As such, in these simulations we have assumed that cases of acquired resistance will be detected only among those who re-present with previously treated disease; the effect of relaxing this assumption is examined in the Discussion. We first compare the incidence-based survey estimate of the proportion of incident disease which is resistant to the calculated proportion resistant among *all* individuals moving into or between TB disease states which includes those who acquire drug resistance during the current course of therapy. We also compare the incidence-based estimate of resistance to the total burden of resistant among prevalent tuberculosis cases calculated at the end of each year.

## Results and Discussion

**Figure 3:**
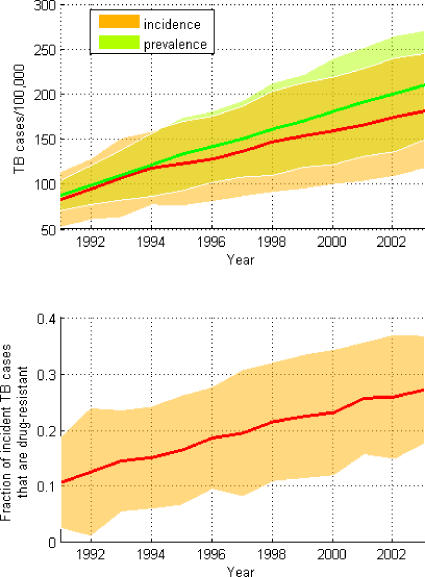
Simulated epidemics. Means and approximate 95% CIs for simulated DR TB epidemics reflecting the incidence (red line, orange shading), prevalence (green line, lime shading), and proportion of disease which is DR (lower subfigure).

The rising trends of TB incidence, prevalence, and drug resistance during the simulated epidemics is shown in Figure 3; the solid and dotted lines depict the mean values for fifty simulated epidemics executed on five different contact networks (each with *n* = 15 and *D* = 5). The shaded areas reveal where 95% of the simulated epidemics fell and reflect variability resulting from both the differences in the realized topology of the contact networks and the inherent stochasticity of the tuberculosis epidemics transmitted on these networks.

**Figure 4:**
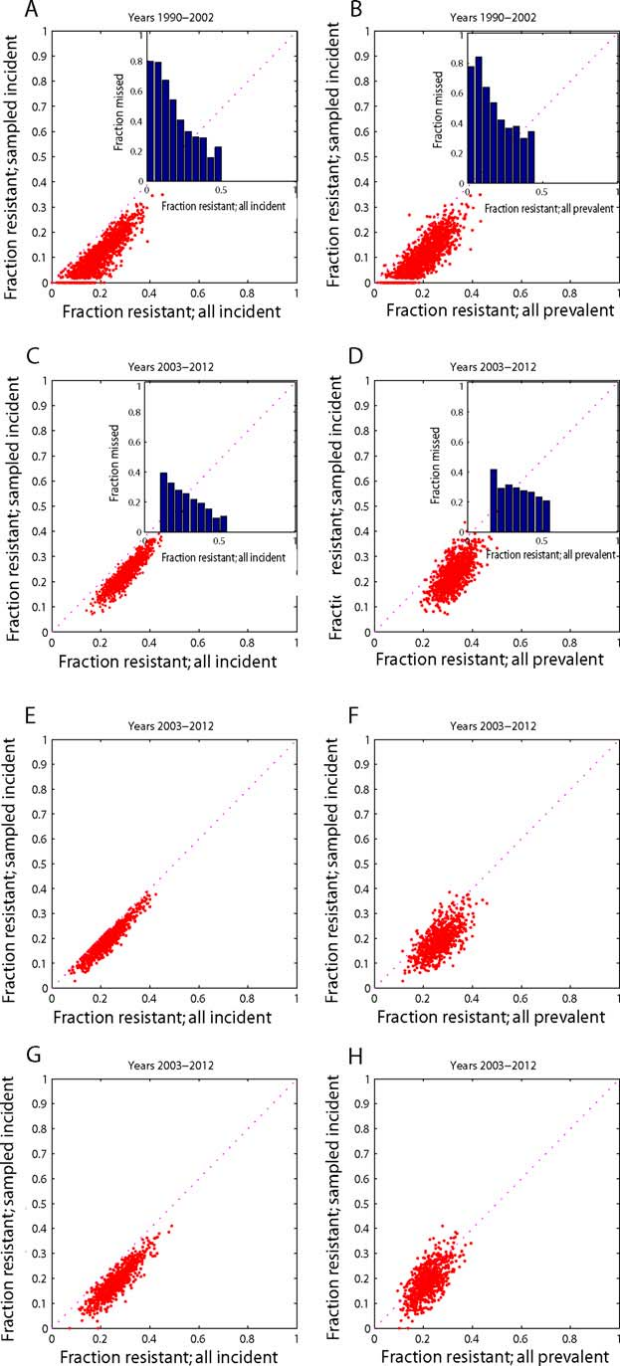
Estimation of burden of resistance based on samples of incident TB cases. The proportion of resistance among incident cases included in surveys (y-axis for all subplots) is graphed against the actual proportion of incident disease which is drug-resistant (x-axis for subplots A, C, E, and G) and against the actual proportion of prevalent disease which is drug-resistant (x-axis for subplots B,D,F, and H). The insets for subplots A–D show the fraction by which the proportion of TB which is DR is underestimated by the survey (y-axis) plotted by the true proportion which is resistant (x-axis). Subplots show the relationship between the surveyed and actual proportions of TB that is resistant during early (A,B) and later periods (C–H) of DR TB epidemics. Three different scenarios are depicted for later periods: no additional interventions to control DR TB (C,D), interventions which limit acquired drug resistance (E,F), and interventions which improve treatment of drug-resistant disease (G,H).

We then simulate the performance of incidence-based sampling and compare the estimated fraction of *sampled* incident cases which are DR to the fraction of *all* incident cases which are DR; in this latter fraction we include all cases of acquired drug resistance in both the numerator and the denominator. Figure 4A shows that incidence-based surveys will underestimate the actual proportion of incident cases that are resistant during the stages of the epidemic when acquired resistance is important. Since DR TB first appears through the acquired route, incidence-based surveys are most prone to underestimate resistance as it is first emerging in a population.

**Figure 5:**
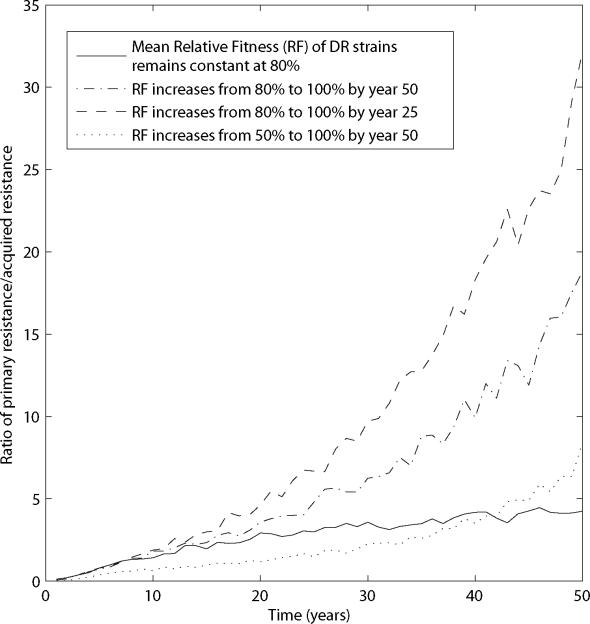
Dominant mechanisms driving resistance change as the epidemic progresses. The mean of the ratio of transmitted to acquired resistance is plotted over 50 years of a simulated epidemic. The rate of increase in this ratio depends on how the relative fitness of resistant strains change over time: four examples are provided, in which the relative fitness of strains remains constant at 80%, increases from 80 to 100% over 50 years, increases from 80 to 100% over 25 years, and increases between 50 and 100% over 50 years, respectively.

As transmission of DR TB becomes an increasingly important mechanism for the continued emergence of resistance, incidence-based surveys produce less biased results. Figure 4C shows substantial reductions in the proportion of incident drug-resistant cases missed by incidence-based sampling during latter stages of an emergent DR TB epidemic. For simplicity in these simulations, we have assumed that the relative transmissibility of the DR strain is fixed at 80% of the transmissibility of the DS strain. In reality, we expect that strain competition would result in selection of increasingly transmissible resistant strains, such that the mean fitness costs of resistance will decrease as the epidemic progresses. While there are not adequate data to describe the time at which an emerging DR TB epidemic switches from being driven by the acquired-resistance mechanism to being driven by transmitted resistance, Figure 5 shows how this transition is dependent upon the distribution of fitness costs associated with resistance. We note that even if fitness costs are fixed (Fig. 5, solid line), the relative importance of transmission increases as resistant strains, initially appearing through inadequate treatment of those with DS TB, become more prevalent. As with other tuberculosis models with similar underlying assumptions about the natural history and parameters, we find that if *all* DR strains suffer total fitness deficits greater than 20 to 30% when compared to DS strains, DR TB does not emerge to become a substantial public health threat.

We also investigate the relationship between the fraction of *sampled* incident TB that is DR, and the fraction of *prevalent* TB in the population that is DR. Since the mean duration of DR TB cases is greater than that for drug-susceptible TB (under the assumption that those infectious with DR strains will not respond as well to standard therapy), the fraction of DR among prevalent disease will be greater than the fraction resistant among incident disease. This enrichment of resistance among prevalent TB cases is present both in early (Fig. 4B) and later phases (Fig. 4D) of the emergence of DR TB; in contrast with the substantial reduction in bias associated with incidence-based sampling as the epidemic progresses, the disparity between resistance among samples of incident and prevalent cases remains relatively stable in these simulations, since the duration of DR TB infectiousness is not affected by the mechanism by which the resistance first appeared.

We find that the introduction of interventions against the emergence of DR TB can alter the relationship between the fraction of sampled incident cases that is DR and both the fraction of all incident cases that is DR and the fraction of prevalent cases that is DR. For example, interventions which reduce acquired resistance, such as the improvement of patient adherence to long and complex drug regimens, can reduce the magnitude of bias in incidence-based estimates of resistance (compare Fig. 4E to Fig. 4C), but may also appear less effective than they really are because the burden of resistance prior to the initiation of the intervention will be underestimated. In contrast, efforts directed at improving the management of DR TB, through the use of drug-sensitivity testing and access to appropriate second-line and third-line antibiotics, do not change the bias in incidence-based estimates of the burden of resistance (compare Fig. 4G to Fig. 4C). As expected, if we reduce the mean duration of DR TB cases to be similar to that of DS TB cases, *e.g.* by instituting control programs which rapidly detect and appropriately treat DR TB, the fraction of incident cases which are resistant becomes a better proxy for the fraction of prevalent cases which are resistant (Fig. 4H).

Our approach, in which we use reduced models of TB epidemics and simplified simulations of drug-resistance surveys, is intended to illustrate potential sources of bias associated with current methods of estimating the burden of drug-resistance from samples of incident TB cases. We use an individual-based model that allows us to directly simulate a sampling process; we can then test for bias by comparing DR TB in our sampled subset to DR TB in the entire population.

The insight we derive from this exercise is qualitative in nature. Since there are several important areas of uncertainty, for example, the manner and degree to which drug-resistance conferring mutations affects the reproductive capacity of the mycobacteria, the rates of acquired resistance during therapy, and the frequency of re-infection and coinfection among others, our ability to make conclusive statements about the magnitude and timing of biases associated with drug resistance surveys conducted among incident TB cases is very limited. Rather than focus on quantifying the amount of bias and specifying the time at which we would expect to see these biases change in the presence and absence of interventions, we have chosen to focus on several key qualitative insights. First, we find that estimates derived from samples of incident TB cases may underestimate the total burden of DR TB. Because individuals acquiring drug resistance while on therapy are less likely to be included in samples of incident cases, the fraction of DR TB among sampled incident cases undercounts resistance among all incident cases. We note that in our results we have assumed that cases which acquire DR TB during their current course of therapy will not be included in samples of incident DR TB; as such, the estimated magnitude of bias associated with these samples will be reduced in settings in which all cases of acquired resistance are captured in these surveys. However, since a portion of these cases of acquired resistance are likely to be missed, this bias will occur, and will be greatest when resistance is first emerging in a population. Thus, we suggest that incidence-based samples may not be adequately sensitive to detect resistance as it first appears in a community. Since the resources needed to limit the spread of resistance in its early stages are substantially less than those needed to address a mature DR TB epidemic, the fact that we may not detect the emergence of resistance in its earliest stages is especially disquieting. Others have argued that estimates of the number of incident cases of DR TB (rather than just the fraction of incident cases which are resistant) is also an important statistic to report [34], [35].

Second, we also demonstrate that resistance among prevalent TB cases will be greater than among incident TB cases if the mean duration of DR TB disease is longer than that of DS TB disease. While it is possible that in some settings co-occurrence of resistance and host factors associated with reduced disease duration (i.e. HIV infection) may actually lead to shorter average durations of disease for those with DR TB, in most situations the DR phenotype will result in failure of standard treatments and a protracted disease course. Since resistance among prevalent cases determines the current resources needed to address extant DR TB in the community, and represents the source of ongoing resistance transmission, estimates of the total burden of DR TB would ideally reflect resistance among prevalent cases. Because studies designed to capture incident cases of acquired DR TB and to measure prevalence of DR TB at the population level may prove too costly and logistically difficult in most settings, we suggest that current estimates from incident based samples should be viewed as a lower bound of the probable burden of resistance.

There are several important factors which are not included in our simple model and which limit the generalizability of our arguments. In particular, we have focused on epidemics of TB in the absence of concurrent HIV co-epidemics. Because HIV infection fundamentally alters the natural history of TB disease among co-infected individuals and changes the transmission dynamics of TB on a population-level, our findings cannot be directly extended to areas in which HIV plays a major role in the emergence of TB and drug resistant TB. We have also not considered the affects of geographic heterogeneity or the role of the private sector in TB treatment, both of which are factors that complicate the measurement of the burden of drug resistant TB; we have discussed the additional challenges posed by these factors elsewhere [36]. The finding that the fraction of incident cases which is drug resistant will be underestimated, especially early in the emergence of drug resistant TB, should hold in situations where acquired drug resistance is how resistance first emerges and where cases of acquired drug resistance may be undercounted. The finding that the fraction of incident cases that is resistant will be less the fraction of prevalent cases which is resistant will apply to areas where the average duration of those with drug resistant disease is longer than for those with drug sensitive disease. Incident-based drug resistance surveys have a very important continuing role in the worldwide assessment of the burden of drug-resistant TB. Because they are relatively easy to implement, they can provide comparable information across many regions of the globe. However, it is important to recognize that these incidence survey approaches do not provide a complete account of the burden of resistance and may, in many circumstances, underestimate the investment in resources that will be required to confront the total burden of resistance within communities.

## Supporting Information

Supplement S1 (0.03 MB DOC)

Table S1 (0.05 MB DOC)

Figure S1 Probability trees demonstrate the order in which the probabilities of events are considered from each of the health/disease states. Parameter explanations and values are provided in the Supplementary Table. The grey box in the upper right-hand corner shows the natural history model structure; the events possible from each of the disease states are linked to this overall model by the letters A through F.
(0.82 MB DOC)

Figure S2 Four hypothetical patient histories demonstrating the time-dependent (relative to infection and re-infection events) rates of progression to active tuberculosis. The height of the bars represents the probability of disease, and the color of the bars correspond to the type of infection (green, drug-sensitive; red, drug-resistant; yellow, mixed). The relative heights of the red and green bars depend on the respective relative fitness of resistant and sensitive strains. Each of these hypothetical individuals is shown to suffer at least one re-infection event to demonstrate that the probability of progression to disease is lower after re-infection than it is for a primary infection, reflecting partial immunity conferred by a previous infection.
(0.64 MB TIF)
