# Supplementary material for: Are Survey-Based Estimates of the Burden of Drug Resistant TB Too Low? Insight from a Simulation Study
Source: PLoS One. 2008 Jun 4;3(6):e2363. doi: 10.1371/journal.pone.0002363 (PMC2408555; doi:10.1371/journal.pone.0002363)
Supplement: Supplement S1 — (0.03 MB DOC) [file pone.0002363.s001.doc]

**Supplement S1**

***State transitions and probability trees***

At each monthly time step, transitions between health/disease states occur stochastically; state transitions occur with the probabilities listed in Table S1, and the sequence in which the probabilities are considered is depicted in the probability trees in Supplemental Figure 1. Since the two strains of TB in the model differ in transmissibility as well as drug-susceptibility, many parameters are subscripted DS or DR to indicate strain.

The interpretation of the probability trees is shown most simply in the tree of an individual in the I_DR_ state (Fig. S1F). The first branch indicates that each month an I_DR_ individual has a ρ_DR_ probability of recovering from disease and transitioning back to the E_DR_ state, and therefore a probability of not recovering equal to (1- ρ_DR_). If the I_DR_ individual does not recover, then he has a μ_DR_ probability of dying from the infection, and a corresponding (1- μ_DR_) probability of not dying. This means that overall, an I_DR_ individual has a probability of dying each month equal to (1- ρ_DR_)*μ_DR_, and not simply μ_DR_. If he does not recover or die, a probability of (1- ρ_DR_)* (1- μ_DR_), then he stays in the I_DR_ state.

The first two branches of the I_DS_ probability tree are analogous to I_DR_ (Fig. S1E). The additional branch at the tip of the tree indicates that the probability of acquiring drug-resistance is equal to (1- ρ_DS_)* (1- μ_DS_)*α. In others words, if an I_DS_ individual does not recover and does not die, then he has a probability of acquiring drug resistance equal to α. For an I_DS_ individual who is not undergoing treatment, α = 0. With this additional factor, the probability of an I_DS_ individual staying in the same state becomes (1- ρ_DS_)* (1- μ_DS_)*(1-α).

There are only slight variations in the other probability trees. For instance, the three-pronged branching in the tree for an E_DR_ individual (Fig. S1C) indicates that if such an individual does not die and does not progress, then he has both a probability φ_DS_ of being infected with drug-sensitive TB and a probability φ_DR_ of being re-infected with drug-resistant TB. A dotted-line indicates a re-infection event. Together this means that the probability that a E_DR_ individual will remain in the E_DR_ state in a given month is equal to (1- μ)*( 1-ρ_DR_)*(1- φ_DS_ - φ_DR_).

***Probabilities of progression from latent states***

Previous studies suggest that the risk of progression from latency to disease is highest within five years of an infection or re-infection event. In our model, we specify the per-month probability of progression for those with a recent first infection (p_1_ primary progression), a recent re-infection (p_3_ exogenous progression), and a more distant infection or re-infection event (p_2_ endogenous progression). Additional complexity arises because individuals can be infected with DS or DR strains (E_DS_, E_DR_) or both strains (E_M_), so that we must index both the timing and the phenotype of infection events. The progression rates arising from latency, and their relation to the timing of infection events, are depicted in Figure S2.

**Supplementary Material References**

1. Rutledge C, Crouch J (1919) The ultimate results in 1,654 cases of tuberculosis treated at the Modern Woodmen of America Sanatorium. Am Rev of Tuberc. 2: 755-6.

2. Blower SM, Gerberding JL (1998) Understanding, predicting and controlling the emergence of drug-resistant tuberculosis: a theoretical framework. J Mol Med. 76: 624-636.

3. Dye C, Williams BG (2000) Criteria for the control of drug-resistant tuberculosis. Proc Natl Acad Sci U S A. 97: 8180-8185

4. Cohen T, Murray M (2004) Modeling epidemics of multidrug-resistant M. tuberculosis of heterogeneous fitness. Nat Med. 10:1117-1121.

5. Gagneux S, Burgos MV, DeRiemer K, Enciso A, Muñoz S, et al. (2006) Impact of bacterial genetics on the transmission of isoniazid-resistant Mycobacterium tuberculosis. PLoS Pathog. 2: e61.

6. Gagneux S, Long CD, Small PM, Van T, Schoolnik GK, Bohannan BJM (2006) The competitive cost of antibiotic resistance in Mycobacterium tuberculosis. Science. 312: 1944-1946.

7. Vynnycky E, Fine PE (1997) The natural history of tuberculosis: the implications of age-dependent risks of disease and the role of reinfection. Epidemiol Infect. 119: 183-201.

8. Dye C, Garnett GP, Sleeman K, Williams BG (1998) Prospects for worldwide tuberculosis control under the WHO DOTS strategy. Lancet. 352: 1886-1891.

9. Springett VH (1971) Ten-year results during the introduction of chemotherapy for tuberculosis. Tubercle. 52: 73-­87.

10. Enarson D, Rouillon A (1994) The epidemiological basis of tuberculosis control. In: Davies P, editors. Clinical tuberculosis. London: Chapman and Hall. pp. 19-32.
