# Supplementary material for: Are Survey-Based Estimates of the Burden of Drug Resistant TB Too Low? Insight from a Simulation Study
Source: PLoS One. 2008 Jun 4;3(6):e2363. doi: 10.1371/journal.pone.0002363 (PMC2408555; doi:10.1371/journal.pone.0002363)
Supplement: Table S1 — (0.05 MB DOC) [file pone.0002363.s002.doc]

**Table S1**. Parameters for surveillance model

| **Parameter** | | **Definition** | **Value^†^** | | **Notes** | | **Source** |
| --- | --- | --- | --- | --- | --- | --- | --- |
| **Birth and death** | | | | | | | |
| **μ** |  | mortality for non-infectious individuals per year | 0.02 | |  | |  |
| **μ_DS_** |  | mortality for untreated I_DS_ individuals per year | 0.25 | |  | | 1 |
| **μ_DR_** |  | mortality for untreated I_DR_ individuals per year | 0.25 | | Assumed to be similar to mortality for I_DS_ | | 1 |
| **γ** |  | birth rate per unoccupied vertex per year | 0.2 | | Fit to maintain stable population size | |  |
| **Infection** | | | | | | | |
| **φ_DS_** |  | rate at which non-infectious individuals are infected with the DS strain per year | | [k_DS_/( k_DS_+ k_DR_)] * 1−[(~φ_DS_)(~φ_DR_)] | | Function of k_DS_ (number of I_DS_ contacts) and τ_DS_ | As below |
| **φ_DR_** |  | rate at which non-infectious individuals are infected with the DR strain per year | | [k_DR_/( k_DS_+ k_DR_)] * 1−[(~φ_DS_)(~φ_DR_)] | | Function of k_DR_ (number of I_DR_ contacts) and τ_DR_ | As below |
|  | **τ_DS_** | Transmission probability per I_DS_ contact per month | 0.12 | |  | | Fit to epidemic |
|  | **τ_DR_** | Transmission probability per I_DR_ contact per month | τ_DR_ = τ_DS_*f | |  | |  |
|  | **f** | Relative fitness of DR strain compared to DS strain | 0.8 | | Affects both infectivity & transmissibility | | 2-6 |
| **Disease progression** | | | | | | | |
| **ν_DS_ ** |  | Rate of progression to DS disease per year | Equal to p_1_, p_2_, or p_3_  (see Figure S2) | | Value depends on the time since infection or re-infection | | As below |
| **ν_DR_ ** |  | DR progression rate per year | Equal to p_1_*f, p_2_*f, or p_3_*f  (see Figure S2) | | Value depends on the time since infection or re-infection | | As below |
|  | **p_1_** | primary DS progression rate (per year) of E_DS_ or E_M_ individual progressing to I_DS_ | 0.03 | | Rate applies for the first 5 years after first infection event | | 7 |
| **Table S1 continued** | | | | | | | |
|  | **p_2_** | endogenous DS progression rate (per year) of E_DS_ or E_M_ individual progressing to I_DS_ | 0.0003 | | Rate applies for the the period after 5 years from an infection or re-infection event | | 7 |
|  | **p_3_** | exogenous reinfection DS progression rate (per year) of E_DS_ or E_M_ individual progressing to I_DS_ | Equal to p_1_*z | | Rate applies for the first 5 years after a re-infection event | | As below |
|  | **z** | partial immunity | 0.4 | | Decreases progression after a re-infection event | | 7,8 |
| **Recovery and treatment** | | | | | | | |
| **ρ_DS_ ** |  | DS recovery rate per year | | 0.85 treated, 0.2 untreated | | Probability of I_DS_ individual recovering to E_DS_ | As below |
| **ρ_DR_ ** |  | DR average recovery rate per year | 0.3 treated, 0.2 untreated | | | Probability of I_DR_ individual recovering to E_DR_ | As below |
|  | **r_SDS_** | DS self-recovery rate per year | 0.2 | |  | | 9,10 |
|  | **r_SDR_** | DR self-recovery rate per year | 0.2 | | Assumed to be similar to self-recovery for I_DR_ | | 9,10 |
|  | **f_T_** | fraction of incident TB cases on therapy | 0.5 | | Model assumption | |  |
| **α** |  | rate of acquiring drug-resistance for I_DS_ individual who is receiving treatment, per month | 0.074 | | Applies only to treatment failures who remain alive | | Fit to epidemic |

^†^these values represent conditional probabilities; the order in which probabilities were considered are shown visually in the probability trees (Supplementary Figure 1) and described in the supplementary text.
