# Supplementary figures and images for: Are Survey-Based Estimates of the Burden of Drug Resistant TB Too Low? Insight from a Simulation Study
Source: PLoS One. 2008 Jun 4;3(6):e2363. doi: 10.1371/journal.pone.0002363 (PMC2408555; doi:10.1371/journal.pone.0002363)

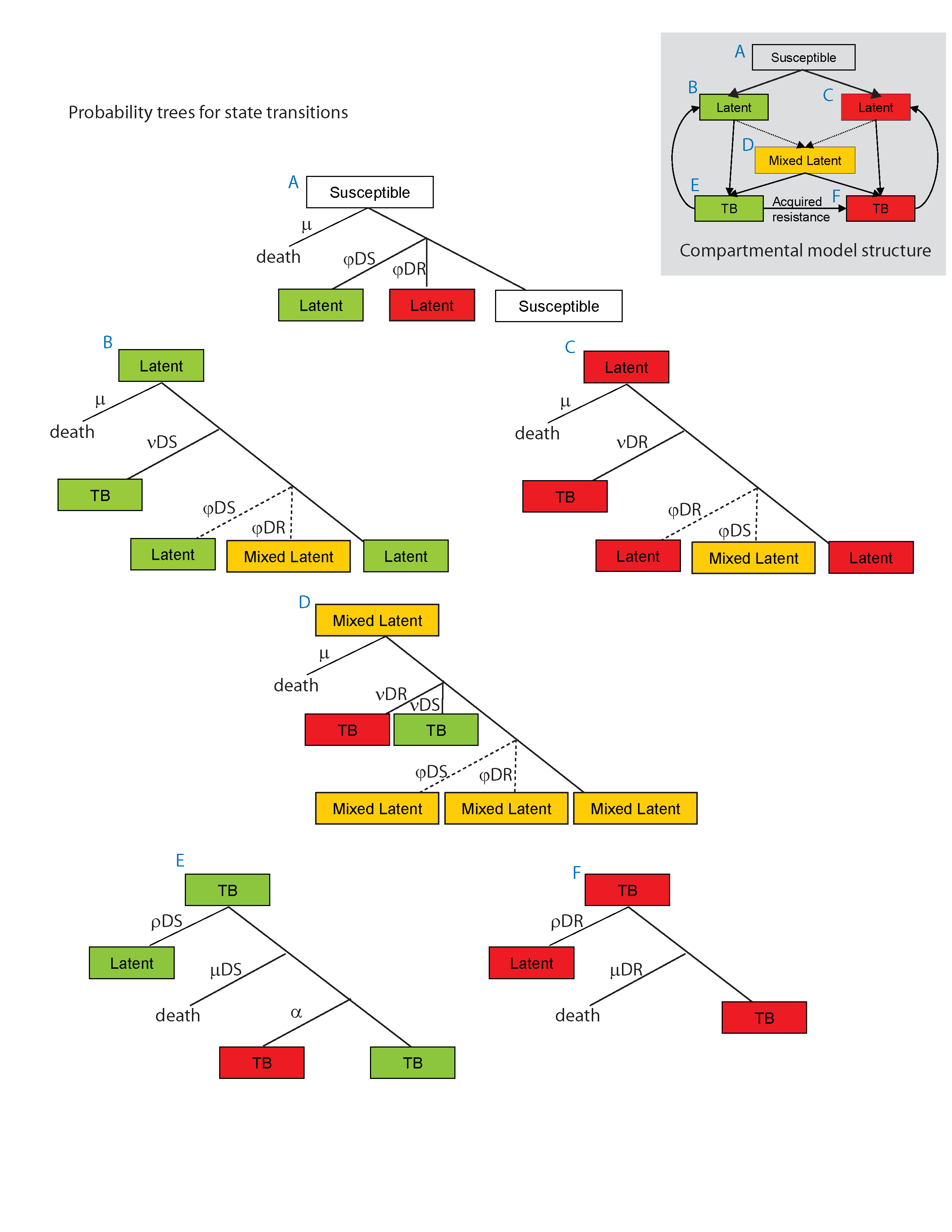

Supplement: Figure S1 — Probability trees demonstrate the order in which the probabilities of events are considered from each of the health/disease states. Parameter explanations and values are provided in the Supplementary Table. The grey box in the upper right-hand corner shows the natural history model structure; the events possible from each of the disease states are linked to this overall model by the letters A through F. (0.82 MB DOC) [file pone.0002363.s003.tif]

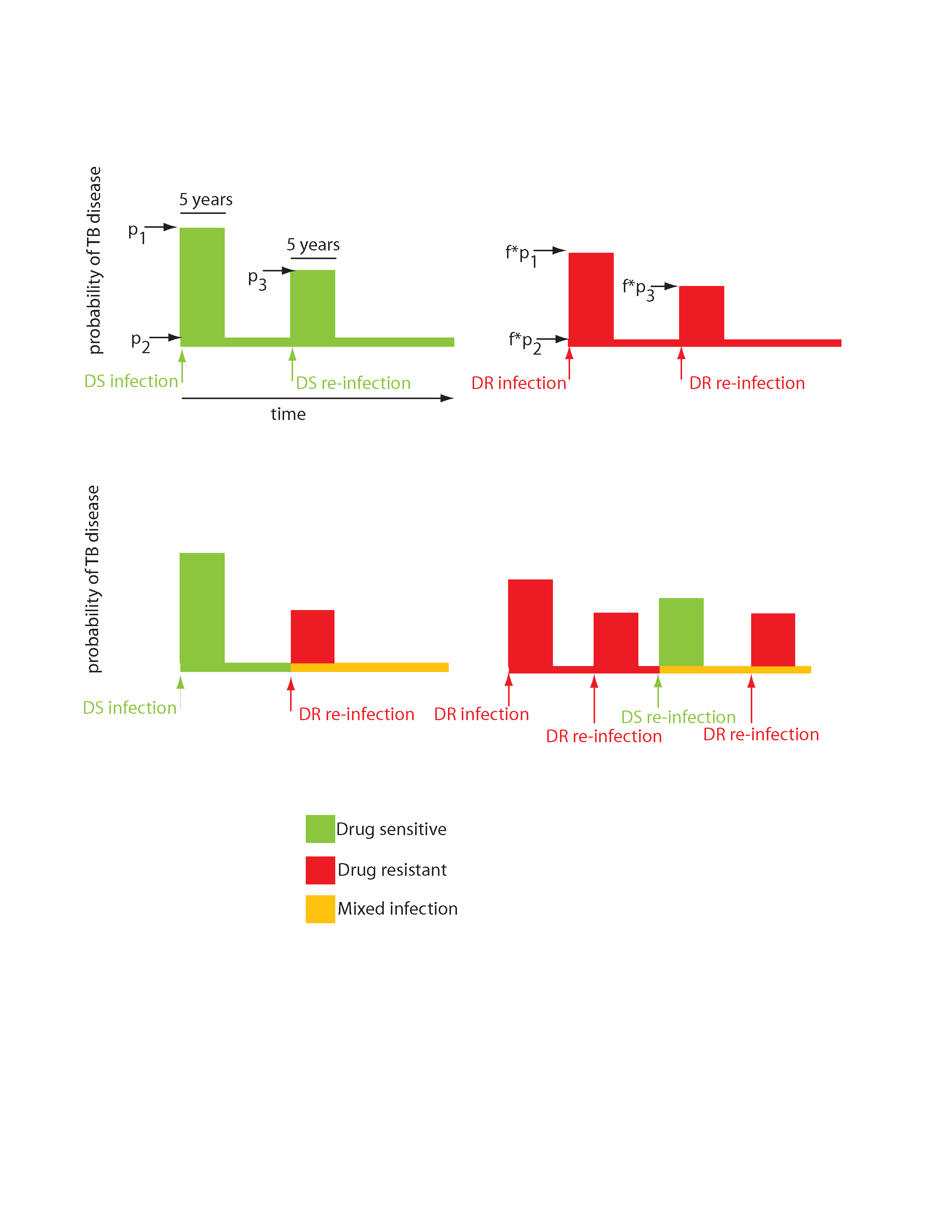

Supplement: Figure S2 — Four hypothetical patient histories demonstrating the time-dependent (relative to infection and re-infection events) rates of progression to active tuberculosis. The height of the bars represents the probability of disease, and the color of the bars correspond to the type of infection (green, drug-sensitive; red, drug-resistant; yellow, mixed). The relative heights of the red and green bars depend on the respective relative fitness of resistant and sensitive strains. Each of these hypothetical individuals is shown to suffer at least one re-infection event to demonstrate that the probability of progression to disease is lower after re-infection than it is for a primary infection, reflecting partial immunity conferred by a previous infection. (0.64 MB TIF) [file pone.0002363.s004.tif]
